# Fluorogenic Membrane Overlays to Enumerate Total and Fecal *Escherichia coli* and Total Vibrionaceae in Shellfish and Seawater

**DOI:** 10.1155/2010/910486

**Published:** 2010-04-12

**Authors:** Gary P. Richards, Michael A. Watson

**Affiliations:** United States Department of Agriculture, Agricultural Research Service, Delaware State University, James W.W. Baker Center, Dover, DE 19901, USA

## Abstract

Three assays were developed to enumerate total and fecal *Escherichia coli* and total Vibrionaceae in shellfish, seawater, and other foods and environmental samples. Assays involve membrane overlays of overnight colonies on nonselective agar plates to detect *β*-glucuronidase and lysyl aminopeptidase activities for *E. coli* and Vibrionaceae, respectively. Cellulose membranes containing the substrates 4-methylumbeferyl-*β*-D-glucuronide (MUG) produced a bright blue fluorescence when overlaid onto *E. coli*, while L-lysyl-7-amino-4-trifluoromethylcoumarin produced green fluorescent foci when overlaid onto Vibrionaceae family members. A multiplex assay was also developed for simultaneously enumerating total *E. coli* and total Vibrionaceae in oysters and seawater. Overall, 65% of overlaid *E. coli* (non-O157:H7) were MUG-positive, compared with 62% as determined by the most-probable-number-MUG assay. The overlays are rapid, simple, and cost effective for quantification purposes. This research provides practical alternatives for monitoring bacterial indicators and potential pathogens in complex samples, including molluscan shellfish.

## 1. Introduction

A technique known as the colony overlay procedure for peptidases (COPP assay) was previously developed as the first test to enumerate total Vibrionaceae in shellfish, seawater, and well water [1, 2]. This assay has been recommended for use in monitoring molluscan shellfish and for potentially regulating their harvest based on the levels of total Vibrionaceae [1]. This total Vibrionaceae approach is similar in concept to traditional sanitary surveys of shellfish harvesting areas, which are based on the total number of fecal coliforms or *E. coli* present in either the shellfish or their surrounding waters [3, 4]. In the original COPP assay, a substrate, L-lysyl-7-amino-4-trifluoromethylcoumarin (L-Lys-AFC), is bound to a cellulose acetate membrane and the membrane is overlaid for 10 minutes onto an overnight culture grown on nonselective agar media. A lysyl aminopeptidase that is present in all Vibrionaceae family members tested to date [5] cleaves the substrate to produce fluorescent foci on the membrane when viewed under longwave UV [1]. In the present study, we extended this overlay concept to produce similar assays for total and fecal *E. coli*. These assays are based on the presence of *β*-glucuronidase (GUD) activity, which is found in most non-O157:H7 *E. coli* and results in cleavage of the substrate 4-methyl-*β*-D-glucuronide (MUG) into the fluorescent 4-methylumbelliferone. MUG-based fluorescence assays for *E. coli* have been previously developed for food and water [6–12]. MUG-based assays of foods and particularly molluscan shellfish often involve the most probable number (MPN) procedure, which is labor intensive, relatively costly, and requires up to three days for results to be obtained. Consequently, simpler, more rapid, less expensive, and more direct enumerative procedures are needed to monitor for total and fecal *E. coli*, particularly in perishable foods, like shellfish. 

In this study, we report on simple membrane overlay methods to separately enumerate total *E. coli* (capable of growing at 37°C), fecal *E. coli *(capable of growing at 44.5°C), and total Vibrionaceae (capable of growing at 37°C), and a fourth multiplex method to enumerate total *E. coli* and total Vibrionaceae simultaneously. These methods are applicable for use in monitoring the presence of these organisms in shellfish and other foods, water, and environmental samples. 

## 2. Materials and Methods

### 2.1. Bacterial Strains

 Stock cultures consisted of 37 strains of non-O157:H7 *E. coli*, as described in Table 1, and 22 Enterobacteriaceae within the genera *Citrobacter, Klebsiella, Pseudomonas, Salmonella, Shigella, Serratia,* and* Yersinia* (Table 2). Forty-three strains of *E. coli* O157:H7 were also evaluated. Most of the strains were derived from the U.S. Department of Agriculture culture collection and represent isolates from food-borne outbreaks. Human pathogenic vibrios consisted of *V. cholerae* O1 and *V. vulnificus* (VV1003), as previously described [5], and *V. parahaemolyticus* O3:K6, a pandemic strain obtained from E. Fidelma Boyd at the University of Delaware, Newark, Delaware.

### 2.2. Shellfish and Seawater

 Eastern oysters (*Crassostrea virginica*) were obtained from a commercial source in Rhode Island and from a tidal river at the University of Delaware Marine Laboratory in Lewes, Delaware. The oysters were naturally contaminated with low levels of *E. coli* and moderate levels of Vibrionaceae. They were collected principally during the warm, summer months when Vibrionaceae were present in the water column. Seawater was also obtained from the University of Delaware Marine Laboratory during the summer.

### 2.3. MPN Assay with MUG

 The MPN procedure using MUG in the EC broth tubes was performed on dilutions of pure cultures and on seawater and oyster homogenates according to the protocol published in the U.S. Food and Drug Administration's Bacteriological Analytical Manual [13]. MUG was obtained from Sigma Chemical Co., St. Louis, Missouri.

### 2.4. MUG Membrane Overlay of Stock Cultures

 The MUG membrane overlay procedure was developed and its specificity was determined using *E. coli* serotypes and other Enterobacteriaceae as listed in Tables 1 and 2. The bacteria were grown in tryptic soy broth (Becton, Dickinson and Co., Sparks, Maryland) supplemented with an additional 0.5% NaCl (1% total NaCl) and were streaked onto 100 mm plates of tryptic soy agar (Becton, Dickinson and Co.) containing a total of 1% NaCl (TSA-N) using a flamed and cooled metal triangle. Plates were incubated overnight (~18 hours) at 37°C. After culture incubation, cellulose chromatography papers (Whatman International, Maidstone, UK) or Whatman no. 1, 4, or 54 cellulose filter paper discs (referred to as cellulose membranes) were cut to the desired size (of the petri dish or colonies to be overlaid) and soaked in 20 mM Tris buffer, pH 8.0 containing 50 mg/L MUG for about 10 seconds. Excess buffer was allowed to drip from the membranes for 5 seconds, and the membranes were overlaid onto colonies growing on the surface of the TSA-N plates. Unused substrate was retained at 4°C for future use and has a shelf life of several weeks. Each membrane was incubated on the plate at 37°C for 30 minutes. Membranes were removed with forceps and placed in empty petri dishes and examined for fluorescent foci using either a hand-held UV lamp at a wavelength of 364 nm or a UV light box at the same wavelength. Fluorescent foci appearing on the membranes were enumerated. Alternatively, membranes could be viewed with the hand-held UV lamp while still overlaying the colonies.

### 2.5. COPP Assay

 The COPP assay was performed with cellulose acetate membranes as previously described [1, 2] or with cellulose chromatography paper (Whatman International). In essence, a substrate L-lysyl-7-amino-4-trifluoromethylcoumarin (L-Lys-AFC; cat. no. AFC-008, MP Biomedicals, Salon, OH) was dissolved in dimethylsulfoxide (DMSO) to 20 mM (2140 *μ*L of DMSO to 25 mg of L-Lys-AFC) and the stock was stored at −20°C until needed. A 250 *μ*M substrate working solution was prepared by combining 25 *μ*L of thawed substrate and 2 mL of 20 mM Tris, pH 9.0. Substrate stock solution may be refrozen and thawed multiple times without appreciable deterioration of the substrate. A cellulose acetate membrane or a plain cellulose membrane (chromatography paper) was cut to the desired size and placed in the working solution for 10–15 seconds. The membrane was lifted with forceps, allowed to drip excess fluid for 5 seconds, and overlaid onto overnight bacterial colonies grown at 37°C on TSA-N plates. Plates were covered and incubated for precisely 10 minutes at 37°C. Each membrane was removed from the plate, placed in a clean petri dish, immediately viewed under longwave (364 nm) UV, and fluorescent foci were counted. Alternatively, the foci could be visualized with a hand held UV lamp while the membrane was still covering the colonies. The appearance of bright white or pale blue fluorescence on cellulose acetate membranes or green fluorescence on cellulose chromatography paper signifies the presence of a lysyl aminopeptidase, which cleaves the substrate. Foci of very weak fluorescence were not counted. Positive controls consisted of *V. cholerae*, *V. parahaemolyticus,* and *V. vulnificus* and were propagated at 37°C. The overlay of Enterobacteriaceae was also performed at 37°C for 10 minutes to determine if any of the species contained a lysyl aminopeptidase activity similar to that found in the Vibrionaceae.

### 2.6. Multiplex COPP/MUG Assay

 A method to simultaneously detect total Vibrionaceae and total *E. coli* was evaluated using cellulose chromatography paper that was soaked in 20 mM Tris, pH 8.0 containing 250 *μ*M L-Lys-AFC and 50 mg/L MUG. Oysters were either 1 : 10 homogenates or dilutions of the homogenates that were prepared in 0.1% peptone buffer. Plates were prepared using 100 *μ*L of each homogenate or dilutions of the homogenates, which were spread over TSA-N plates with a flamed and cooled metal triangle. After overnight incubation at 37°C, membranes containing both substrates were overlaid onto the culture plate. Vibrionaceae were enumerated in situ after 10 minutes followed by the enumeration of MUG-positive (GUD-positive) colonies after an additional 20 minutes (30 minutes total). It was anticipated that total *E. coli* could be easily distinguished from total Vibrionaceae based on the color of the fluorescent foci.

### 2.7. Beta-Glucuronidase in *E. coli* O157:H7

 Forty-three principally food-borne outbreak strains of *E. coli* O157:H7 were screened for GUD activity by both the MPN-MUG assay [13] and by the MUG overlay of colonies that were grown on TSA-N and incubated at 37°C for 2 hours followed by 44.5°C overnight. Plates were overlaid for 30 minutes and the membranes were viewed on a UV light box. The fluorescent foci were enumerated.

### 2.8. Screening of Oysters and Seawater

 Oysters and seawater were screened by the membrane overlay assay for total *E. coli *and total Vibrionaceae and by multiplex assays for both by spread plating 100 *μ*L of a 1 : 10 or 1 : 100 oyster homogenate, prepared in 0.1% peptone buffer, or 100 *μ*L of undiluted seawater onto TSA-N plates. The plates were allowed to incubate at either 37°C for total *E. coli* and total Vibrionaceae overlays or at 37°C for 2 hours followed by 44.5°C overnight for *E. coli* overlays using cellulose membranes containing MUG and COPP separately and combined. Samples were occasionally plated in duplicate and one plate incubated at 37°C for the multiplex assay of total Vibrionaceae and total *E. coli* while the other plate was incubated at 37°C for 2 hours and then at 44.5°C overnight for fecal *E. coli* enumeration.

## 3. Results

### 3.1. Optimization of Membrane Fluorescence

 Initial tests showed that overnight colonies of *E. coli* growing on TSA-N plates could be overlaid with cellulose membranes (chromatography paper or Whatman no. 1, 4, or 54 filter paper discs) soaked in MUG to produce strong blue fluorescent foci on the membranes, corresponding with the site of contact between the colony and the membrane. Comparable results were obtained regardless of which type of membrane was used. Consequently, we selected the chromatography paper to perform MUG overlays throughout this study. An evaluation of substrate concentrations showed that strong fluorescence intensity was achieved when membranes were saturated in ≥50 mg/L MUG. Membranes were incubated for 15−60 minutes, and then viewed for fluorescent foci in situ with a hand-held UV lamp, or the membrane could be removed to a petri dish and viewed with the hand-held UV lamp or placed on a UV light box for viewing. Larger colonies generally required less time for fluorescence development. Optimal results were obtained after a 30–60-minute overlay and 30 minutes was selected for the remainder of the study. In contrast, colonies to be screened for total Vibrionaceae by the COPP assay were saturated in 250 *μ*M of L-Lys-AFC and overlaid for only 10 minutes to preclude weak enzyme activity, which is present in some Enterobacteriaceae and other non-Vibrionaceae, from producing false positive fluorescent foci.

**Figure 1:**
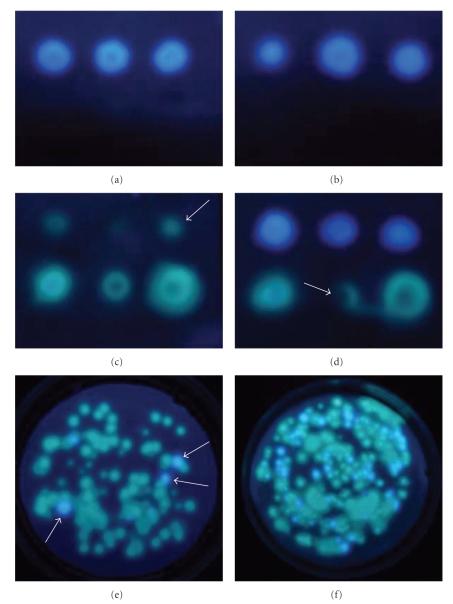
Examples of representative fluorescent foci from membrane overlays of overnight colonies on plates of tryptic soy agar containing 1% NaCl. (a–d) represent plates spotted with *E. coli* and vibrios, incubated overnight, and then overlaid with membranes saturated with fluorescent substrate(s). Specific bacterial strains in panels (a–d) are as follows: (left to right, top row), *E. coli* serotypes O5:NM, O9:H21, and O22:H8; (left to right, bottom row), *V. cholerae* O1, *V parahaemolyticus* O3:K6, and *V. vulnificus* VV1003. Individual panels are as follows: (a) MUG assay for total *E. coli* (blue fluorescent foci) with incubation at 37°C overnight, (b) MUG overlay for fecal *E. coli* after incubation at 37°C for 2 hours followed by 44.5°C overnight, and (c) COPP overlay for total Vibrionaceae (green fluorescent foci) after overnight incubation at 37°C. The arrow signifies weak fluorescence that is occasionally observed in *E. coli* and other bacteria due to the presence of small quantities of lysyl aminopeptidases and is especially noticeable if overlays exceed 10 minutes. (d) Multiplex assay for total *E. coli* (blue) and total Vibrionaceae (green) after overnight incubation at 37°C. Arrow depicts a *V. parahaemolyticus* colony which displays weak fluorescence due to the unintentional sticking of most of the colony to the membrane and subsequent blockage of UV light penetration. (e and f) Multiplex assay for total *E. coli* and total Vibrionaceae performed on cultures of oyster homogenate naturally contaminated with a low level (e) and a high level (f) of *E. coli* after overnight incubation at 37°C. Arrows in (e) indicate three of the four blue fluorescent foci representing *E. coli*. All overlays were performed with cellulose membranes.

 Figures 1(a)–1(d) show MUG and COPP fluorescence on cellulose membranes after overlaying colonies of three serotypes of non-O157: H7 *E. coli* (top row in each panel) and three Vibrionaceae (bottom row in each panel). Edges of the foci are not distinct and sharp, but somewhat fuzzy because enzyme activity is via diffusion of the enzymes within the membranes. Examples of representative MUG fluorescence on the membranes are shown for total *E. coli* that had been incubated at 37°C overnight and overlaid for 30 minutes with MUG-containing membranes (Figure 1(a)), and for fecal *E. coli* grown at 37°C for 2 hours, incubated overnight at 44.5°C, and overlaid for 30 minutes with a MUG membrane (Figure 1(b)). Three *Vibrio* spp. are on the bottom rows and are MUG negative (nonfluorescent). These same vibrios are strongly COPP positive after overnight incubation at 37°C and overlay for 10 minutes with L-Lys-AFC, producing green fluorescence on cellulose membranes, as depicted in Figure 1(c). Very weak COPP fluorescence is occasionally observed in the *E. coli* (arrow, Figure 1(c)), and such foci should not be counted as Vibrionaceae because of the weak signal. This fluorescence may be minimized by enumerating the foci immediately after the 10-minute overlay. Results of the multiplex assay on cultures incubated at 37°C overnight, followed by a 30-minute overlay with MUG and L-Lys-AFC (combined) are shown in Figure 1(d) with blue fluorescence from the *E. coli* on the top row and green fluorescence from the vibrios on the bottom row. The arrow in Figure 1(d) indicates the blockage of UV light from an opaque colony of *V. parahaemolyticus* which inadvertently transferred to the membrane and blocked the passage of the UV light from the light box through the membrane. Such transfer of opaque colonies to the membranes occasionally occurs and appears to be a function of the stickiness of the colony. In such cases, foci may be readily observed by holding a hand-held UV lamp on the opposite side of the membrane (the side without the opaque material).

### 3.2. Comparison of MUG Overlays and MPN-MUG Assays of Non-O157:H7 *E. coli*

 The MUG overlay and the MPN-MUG assays were compared for 37 strains of *E. coli* (non-O157:H7). Twenty-four (65%) of the 37 strains were positive by the MUG overlay and 23 (62%) were positive by the MPN-MUG assay; however, four colonies that were MUG-negative by overlay were positive or weakly positive by the MPN-MUG method, specifically strains O14:NM, O42:H2, O103:H2, and O111:NM (Table 1). Likewise, five isolates that were MUG-negative according to the MPN-MUG method were positive by the overlay method, specifically one each of the O4:NM and O26:H11 strains, and O78:H11, O91:H, and O91:H14 (Table 1). MPN-MUG often gave weak fluorescence signal (listed as ± in Table 1). Repeat assays of these isolates showed variability in the perceived fluorescence intensities for those cultured in MPN-MUG media. No variability was seen on the overlays, which consistently produced bright blue foci. COPP overlays of these cultures were performed for 10 minutes with readings taken immediately after overlay, and weakly positive results were obtained for four of the isolates (Table 1).

### 3.3. Comparison of MUG Overlays, MPN-MUG Assays, and COPP Assays for Non-*E. coli* Enterobacteriaceae

 Twenty two non-*E. coli* Enterobacteriaceae (*Citrobacter* spp., *Klebsiella pneumonia*, *Pseudomonas* spp., *Salmonella* spp, *Shigella* spp., *Serratia liquefaciens*, and *Yersinia enterocolitica*) were compared using the overlay and MPN-MUG procedures. Results are listed in Table 2. COPP assays were also performed. Differentiation of the *Shigella* spp. was possible using individual MUG and COPP assays where one *Sh. boydii* was both MUG and COPP positive, *Sh. flexneri* was both MUG and COPP negative, *Sh. dysenteriae* was MUG negative and COPP positive, and *Sh. sonnei* was MUG positive and COPP negative with cultures propagated at 37°C (Table 2). *Shigella boydii*, *Sh. dysenteriae*, and *Y. enterocolitica* were COPP positive indicating an enzyme analogous to the lysyl aminopeptidase that was found in Vibrionaceae family members. This is the first time we detected strong COPP positive colonies associated with bacteria that were unrelated to the Vibrionaceae. *Pseudomonas aeruginosa* produced an orange fluorescence and was considered COPP and MUG negative.

### 3.4. Screening for *β*-Glucuronidase in *E. coli* O157:H7

 Forty-three *E. coli* O157:H7 strains were tested in triplicate and were MUG negative by both the overlay and MPN-MUG assays (data not shown). It is well known that O157:H7 strains lack GUD activity and are therefore MUG negative [14] with few exceptions [15].

### 3.5. Multiplex Membrane Overlay for Total *E. coli* and Vibrionaceae in Oysters and Seawater

 Membranes simultaneously soaked in MUG and L-Lys-AFC were used in a multiplex format to detect total *E. coli* and total Vibrionaceae on plates that were grown overnight at 37°C (Figures 1(e) and 1(f)). In the multiplex assay of oyster homogenates, green and dark blue foci are obtained for Vibrionaceae and total *E. coli*, respectively, when incubated at 37°C (Figures 1(e) and 1(f)). For accurate enumeration, fluorescent foci must be counted in stages. Membranes were incubated for 10 minutes on the plates and viewed with a hand-held UV. Green fluorescent foci, representing the Vibrionaceae colonies, were counted immediately, to avoid low levels of enzymes in some non-Vibrionaceae from leading to false positive results. The plate was then incubated for an additional 20 minutes or longer at 37°C for blue fluorescence development, which is indicative of colonies of total *E. coli*. For illustration purposes only, membranes shown in Figures 1(e) and 1(f) were illuminated on a UV light box and photographed after a 30-minute overlay, which was sufficient to give bright blue fluorescence from the GUD-positive colonies but was longer than recommended for accurate Vibrionaceae enumeration. Simultaneous detection is only practical if countable levels of total *E. coli* and total Vibrionaceae are present on the same dilution plate, although Figures 1(e) and 1(f) demonstrate the ease with which total *E. coli* colonies may be identified, even on crowded plates. Multiplex assays are not appropriate for cultures incubated at 44.5°C, since many Vibrionaceae may not grow at elevated temperatures. One exception was our *V. cholerae* O1 which produced typical COPP fluorescence when grown at 44.5°C.

### 3.6. MUG Overlays at 44.5°*C* for *E. coli* in Oysters

 If total Vibrionaceae and total and fecal *E. coli* enumeration are desired for a sample, duplicate plating allows one plate to be incubated at 37°C for total Vibrionaceae and total *E. coli* while the other plate is incubated at 37°C for 2 hours and then at 44.5°C overnight for fecal *E. coli* enumeration. The 2 hours incubation at 37°C constitutes a resuscitation step, as oysters may contain stressed bacteria which need to be resuscitated. Higher *E. coli* counts were obtained in our assays when the resuscitation step was employed. From a safety standpoint, the MUG assay offers additional protection against pathogens, by detecting some strains of *Salmonella* and *Shigella* which were MUG positive and could grow at 44.5°C, specifically, *S. *Montevideo, *Sh. sonnei*, and one of two strains of *Sh. boydii* (Table 2).

### 3.7. Seawater Analyses

 Seawater produced bacterial colonies that gave a fluorescence signal comparable to that found in oyster homogenates for total and fecal *E. coli* and total *Vibrionaceae*; however, the levels of bacteria were generally 10- to 100-fold less than in filter-feeding molluscan shellfish, which bioconcentrate bacteria from the seawater within their edible tissues.

## 4. Discussion

By definition, fecal coliforms, including fecal *E. coli*, are Gram-negative bacteria which ferment lactose with the production of acid and gas at 44.5−45.5°C. Such organisms are indicative of human gut bacteria and can be used as indicators for the possible presence of human fecal pathogens in foods and water. The MPN procedure capitalizes on the use of lactose-containing media, the collection of gas, and growth at 44.5−45.5°C, so that positive isolates meet the classical definition for fecal coliforms. MPN-MUG procedures use MUG as an indicator for the presence of *E. coli*, since *E. coli* are the primary producers of GUD at these elevated temperatures; therefore, positive MPN results in the presence of MUG are considered confirmatory for the presence of *E. coli* [13]. Current MPN assays for total and fecal coliforms, or *E. coli*, are tedious, costly, and time consuming. The incorporation of MUG into EC broth for the MPN detection of *E. coli* [8] has hastened the analysis over previous MPN methods but is still complex and produces false positives which can compromise results. According to the U.S. Food and Drug Administration's Bacteriological Analytical Manual [13], the MPN-MUG assay requires the use of new borosilicate glass tubes as well as new gas collection (Durham) tubes and the tubes must be prescreened for fluorescence, as some tubes exhibit autofluorescence. In our hands, autofluorescence was a problem and may have contributed to the weakly positive MPN-MUG results for *E. coli* O14:NM, O42:H2, O103:H2, and O111:NM, which were consistently negative by the MUG overlay procedure (Table 1). In the United States, oysters and their growing waters are monitored for fecal coliforms or *E. coli* using a 5-tube MPN approach, which means that 15–30 or more new tubes may be required for a single analysis, depending on the number of dilutions required and the number of presumptive positive tubes that need to be transferred from lauryl sulfate tryptose broth to EC broth, thus making this procedure costly and impractical for routine use.

Since one of the major obstacles in monitoring for environmental contamination and food safety is the lack of practical and cost effective assays, our goal was to produce simple, more rapid, and inexpensive procedures to enhance monitoring efforts. This paper provides new tools to monitor for total and fecal *E. coli* and total Vibrionaceae levels. In this study, we developed a simple plate cultivation and MUG-membrane overlay technique to detect bacteria that are likely fecal *E. coli* based on GUD production and their ability to grow at 44.5°C. For our overlay procedure for total *E. coli*, we recognize that occasional pathogens other than *E. coli* may be GUD positive, like some Salmonella and Shigella strains (Table 2), or some Streptococci [16]; however, the detection of other occasional pathogens simply enhances the benefits of using this test as a potential regulatory tool. These techniques may be applicable to virtually any food or environmental sample. Endogenous levels of GUD, which are high in some fish and shellfish [8, 17], are not a problem in our plate-based membrane overlays, since the overnight incubation of the plates at either 37°C or 44.5°C degrades endogenous, tissue-specific enzymes well before the plate is overlaid with the membrane containing the fluorogenic substrate. Consequently, in the many assays we performed on oysters, we saw no background fluorescence, even though oyster tissues are known to contain GUD [8].

In addition to the complexities of performing traditional MPN-based assays, analyses for Vibrionaceae in seawater and shellfish are usually complex and are generally limited to picking a few isolates from a plate for complex biochemical or molecular biological testing for specific pathogenic strains. Monitoring of shellfish or seawater for pathogenic vibrios has failed to stop outbreaks of *V. parahaemolyticus*, while routine monitoring programs for *V. vulnificus* are nonexistent in spite of the fact that *V. vulnificus* in oysters causes occasional illness and death. Acceptable baseline or threshold levels of Vibrionaceae and total or fecal *E. coli* should be determined for implementation into regulations for harvesting or distribution of shellfish. Threshold levels currently exist for coliforms in harvesting area waters and shellfish meats [3, 4] but are based on MPN approaches. No methods have been established to date for regulation of shellfish harvesting or distribution based on total Vibrionaceae levels or *E. coli* levels using simple overlay methods. 

In screening 37 strains of non-O157:H7 *E. coli* that were associated with major foodborne outbreaks of illness, 24 (65%) were positive by the MUG overlay and 23 (62%) were positive by the MPN-MUG assay. This is consistent with a study by Chang et al. [18], who showed 66% GUD positivity from *E. coli* isolated from healthy individuals. In contrast, other studies showed the recovery of GUD in 95.5% [19], 96.5% [20], and 91% [8] of the isolates examined. Potential reasons for these differences in GUD distribution among the isolates were suggested by Chang et al. [18], but there have been no conclusive studies explaining such variability. In addition to a subset of non-O157:H7 isolates that were MUG negative, 43 isolates of *E. coli* O157:H7 that were derived from a wide variety of food-borne outbreaks were MUG negative. The failure to detect O157:H7 strains is a limitation of both MPN-MUG and the MUG overlay methods. This finding does not lessen the importance of the MUG membrane overlay procedure, since O157:H7 strains constitute a minority of the *E. coli* in environmental isolates and other MUG-based cultural assays, like the U.S. Food and Drug Administration-recommended MPN-MUG assay for *E. coli,* also fail to detect the O157:H7 serotype [13].

*Escherichia coli* O157:H7 strains do not contain enzymatically active GUD, although these strains contain the *gusA* (*uidA*) gene, which encodes GUD and two regulatory regions [21]. There does not appear to be regulatory repression to restrict GUD formation, since antibody against GUD detects the enzyme; however, the GUD is in an inactive form lacking hydrolytic activity [21]. The cause for this inactive protein is the presence of a guanosine-guanosine insertion causing a frameshift resulting in the introduction of a premature termination codon in the *gusA* gene in all strains of *E. coli* O157:H7 tested, but not in other serotypes [22]. This mutation accounts for the presence of an incomplete protein lacking GUD activity; therefore, MUG-based assays are not appropriate for the detection of O157:H7 isolates. 

 Advantages of the overlays are that they are far simpler, less expensive, and directly quantitative, unlike MPNs which give statistical probabilities of counts. Culturing bacteria on nonselective TSA-N also has an advantage over selective media, like McConkey agar with or without MUG, since selective media are often inhibitory to environmentally stressed bacteria. With multiplex overlays, we have consolidated assays to simplify monitoring in order to drive down costs for screening and to promote more frequent testing for total *E. coli* and Vibrionaceae. Both the MUG overlay and the COPP assays are relatively fast, with results derived within 24 hours. MPN assays require up to 3 days for completion (24–48 hours incubation in LST broth followed by 24 hours in EC broth) and additional time if *E. coli* confirmation via biochemical testing is desired. Together, the MUG and COPP overlay methods provide new options for quantifying total and fecal *E. coli* and Vibrionaceae in seawater, shellfish, and other food and environmental matrices. 

Unlike the original COPP assay, which used cellulose acetate membranes that could be prepared in advance, dried for storage, and soaked in buffer before overlay [1, 2], the cellulose membranes used for COPP and MUG assays in this paper cannot be prepared in advance or dried. They must be made immediately before use. This is because COPP substrate covalently bonds to cellulose acetate membranes, but not to plain cellulose membranes, and MUG does not bind to either membrane. Rewetting membranes containing MUG would wash away the substrate. Although we used cellulose acetate and plain cellulose membranes to screen stock cultures for lysyl aminopeptidase activity, we used cellulose membranes exclusively for multiplex assay for total coliforms and total Vibrionaceae. This was because COPP assays on cellulose acetate membranes produced a bright light blue fluorescence while MUG produced a stronger blue fluorescence; thus, differentiation of the two colors was difficult at times. It became clear that the green and blue fluorescence obtained on the plain cellulose membranes was easier to discriminate between than blue and light blue fluorescence on the cellulose acetate membranes. Multiplex overlays for the simultaneous detection of both total *E. coli* and total Vibrionaceae may be performed when both groups of bacteria are in relatively the same proportion; that is, both can be enumerated from the same dilution plate. 

Methods to monitor molluscan shellfish harvesting and distribution, much like methods for other foods, water, or environmental samples, are often outdated and out of touch with today's needs. It is necessary to shift analytical and regulatory paradigms to simpler, more practical, and cost effective measures to monitor relative levels of contamination. Given the development of simple membrane overlay assays, we propose the establishment of membrane overlay-based monitoring for total and fecal *E. coli* and total Vibrionaceae. For routine monitoring purposes, it may be time to shift from a search for specific *Vibrio* pathogens to the enumeration of total Vibrionaceae. Environmental factors responsible for increases in human pathogenic vibrios within shellfish or their growing waters will likely cause an increase in the levels of many other Vibrionaceae family members, including pathogenic strains; therefore, the COPP assay could prove useful in regulating shellfish harvesting based on total Vibrionaceae counts [1]. This approach is consistent with U.S. and European standards for shellfish harvesting, where coliform levels are currently used to regulate shellfish harvesting and distribution [3, 4]. In an aquaculture setting, the simple COPP assay may serve as an index of fish health and disease, where spikes in Vibrionaceae levels over what is normal and customary would suggest a need for more refined assays for specific pathogens and for corrective actions to be implemented within the aquaculture or hatchery setting. Membrane overlays are simple and practical for routine monitoring and could enhance food safety by allowing increased numbers of samples to be tested at a reduced cost or by providing a simple alternative to procedures that are too rigorous for routine use.

## Figures and Tables

**Table 1:** *Escherichia coli* isolates (non-O157:H7) used in this study and results of 4-methyl-*β*-D-glucuronide (MUG) overlay for the detection of *β*-glucuronidase-producing bacteria, most probable number (MPN) assay in EC tubes containing MUG, and colony overlay procedure for peptidases (COPP) overlay for lysyl aminopeptidase activity.

					
Serotype	ID code	Source^1^	No. MUG+ in triplicate overlays^2^	MPN-MUG tubes^3^		
				Growth	Gas	MUG fluorescence	
O4:NM	88.0545	USDA	3	+	+	±
O4:NM	90.2258	USDA	3	±	−	+
O4:NM	STEC	USDA	3	+	+	−
O5:NM	79.0396	USDA	3	+	+	±
O5:NM	84.0166	USDA	0	+	+	−
O5:NM	85.0587	USDA	3	+	+	±
O9:H21	83.0625	USDA	3	+	+	±
O14:NM	STEC	USDA	0	±	−	±
O22:H5	95-3322 STEC	USDA	3	+	+	+
O22:H8	90-0327	USDA	3	+	+	±
O26:H11	88.1457	USDA	3	+	+	−
O26:H11	90.0105	USDA	3	+	+	+
O26:H11	3359-70	USDA	3	+	+	±
O28ac:H-	CVD/EI-1	USDA	3	+	−	+
O28ac:H-	CVD/EI-2	USDA	0	+	−	−
O28ac:H-	CVD/EI-4	USDA	3	+	−	+
O29:NM	43982	ATCC	0	+	+	−
O38:H21	96-3307 CDC	USDA	3	+	+	+
O42:H2	88.0501	USDA	0	+	+	±
O44:H18	O42	USDA	3	+	+	+
O78:H11	35401	ATCC	3	+	−	−
O78:K80:H12	34896	ATCC	3	+	+	+
O91:H	3610606 Stx2	USDA	3	+	+	−
O91:H14	96.0348Stx1	USDA	3	+	+	−
O103:H2	87.1368	USDA	0	+	+	±
O103:H2	93.0626	USDA	3	+	+	±
O103:H2	97-3112 STEC	USDA	0	+	+	−
O103:H25	97-3112 CDC	USDA	0	+	+	−
O111:H-	JB1-95	USDA	0	+	−	−
O111:NM	91.1030	USDA	0	+	+	±
O128:H45	19-3305 CDC	USDA	0	+	+	−
O128:H45	96-3305 STEC	USDA	0	+	+	−
O137:H41	88-3493 CDC	USDA	3	+	+	+
O145:NM	83-75 STEC	USDA	3	+	+	+
O145:NM	87.1009	USDA	3	+	+	+
O157:NM	21902B	USDA	0	+	+	−
O:H4	06E01767	USDA	3	+	+	+

^1^Abbreviations: ATCC: American Type Culture Collection, Manassas, Virginia; USDA: United States Department of Agriculture, Agricultural Research Service, Wyndmoor, Pennsylvania.

^2^MUG and COPP overlays were performed at 37°C.

^3^MPN-MUG assays were incubated at 44.5°C.

**Table 2:** Other Enterobacteriaceae used in this study and the results of 4-methyl-*β*-D-glucuronide (MUG) overlay for the detection of *β*-glucuronidase-producing bacteria, most probable number (MPN) assay in EC tubes containing MUG, and colony overlay procedure for peptidases (COPP) overlay for lysyl aminopeptidase activity.

Pathogen	ID code	Source^1^	No. MUG+ in triplicate overlays^2^	MPN-MUG tubes^3^			COPP overlay^2^
				Growth	Gas	MUG fluorescence	
*Citrobacter braakii*	43162	ATCC	0	−	−	−	−
*Citrobacter freundii*	8090	ATCC	0	−	−	−	−
*Citrobacter freundii*	33128	USDA	0	−	−	−	−
*Klebsiella pneumonia*	13883	ATCC	0	−	−	−	−
*Pseudomonas aeruginosa*	7700	ATCC	0	−	−	−	–
*Pseudomonas fluorescens*	43892	ATCC	0	−	−	−	−
*Salmonella *Dublin	15480	ATCC	0	±	−	−	−
*Salmonella *Enteritidis	SAL 196 H 3502	USDA	0	±	−	−	−
*Salmonella *Montevideo	SAL 2#61 FSIS 051	USDA	3	±	−	+	−
*Salmonella *Newport	SAL1#86 H 1073	USDA	0	±	−	−	−
*Salmonella *Typhimurium	G 7601 WI U 302	USDA	0	±	−	−	−
*Salmonella *Typhimurium	H 3380 CA DT 104	USDA	0	±	−	−	−
*Salmonella *Typhimurium	SAL 2#46 FSIS 026	USDA	0	±	−	−	−
*Serratia liquefaciens*	35551	ATCC	0	−	−	−	−
*Shigella boydii*	9207	ATCC	2	−	−	−	−
*Shigella boydii*	BS512	USUHS	3	±	−	+	+
*Shigella dysenteriae*	BS506	USUHS	0	−	−	−	+
*Shigella flexneri*	2457T	USUHS	0	−	−	−	−
*Shigella flexneri*	12022	ATCC	0	−	−	−	−
*Shigella sonnei*	BS514	USUHS	3	±	−	+	−
*Shigella sonnei*	25931	ATCC	3	±	−	+	−
*Yersinia enterocolitica*	O:8, 12#08	USDA	0	−	−	−	+

^1^Abberviaitons: ATCC: American Type Culture Collection, Manassas, Virginia; USDA: United States Department of Agriculture, Agricultural Research Service, Wyndmoor, Pennsylvania; USUHS: Uniformed Services University of the Health Sciences, Bethesda, Maryland.

^2^MUG and COPP overlays were performed at 37°C.

^3^MPN-MUG assays were incubated at 44.5°C.
